# Genetic architecture dissection by genome-wide association analysis reveals avian eggshell ultrastructure traits

**DOI:** 10.1038/srep28836

**Published:** 2016-07-26

**Authors:** Zhongyi Duan, Congjiao Sun, ManMan Shen, Kehua Wang, Ning Yang, Jiangxia Zheng, Guiyun Xu

**Affiliations:** 1 National Engineering Laboratory for Animal Breeding and MOA Key Laboratory of Animal Genetics and Breeding, College of Animal Science and Technology, China Agricultural University, Beijing, 100193, China; 2 Jiangsu Institute of Poultry Science, Yangzhou, Jiangsu, 225125, China

## Abstract

The ultrastructure of an eggshell is considered the major determinant of eggshell quality, which has biological and economic significance for the avian and poultry industries. However, the interrelationships and genome-wide architecture of eggshell ultrastructure remain to be elucidated. Herein, we measured eggshell thickness (EST), effective layer thickness (ET), mammillary layer thickness (MT), and mammillary density (MD) and conducted genome-wide association studies in 927 F_2_ hens. The SNP-based heritabilities of eggshell ultrastructure traits were estimated to be 0.39, 0.36, 0.17 and 0.19 for EST, ET, MT and MD, respectively, and a total of 719, 784, 1 and 10 genome-wide significant SNPs were associated with EST, ET, MT and MD, respectively. ABCC9, ITPR2, KCNJ8 and WNK1, which are involved in ion transport, were suggested to be the key genes regulating EST and ET. ITM2C and KNDC1 likely affect MT and MD, respectively. Additionally, there were linear relationships between the chromosome lengths and the variance explained per chromosome for EST (R^2^ = 0.57) and ET (R^2^ = 0.67). In conclusion, the interrelationships and genetic architecture of eggshell ultrastructure traits revealed in this study are valuable for our understanding of the avian eggshell and contribute to research on a variety of other calcified shells.

Avian eggshell is a biphasic composite of organic matrix and inorganic minerals and provides the basic mechanical protection, gas exchange and calcium source for embryonic development^1^. The unique structure of the avian eggshell also plays a vital role in preventing microbial contamination of the egg contents and in reducing economic losses due to the breakage of eggs in the poultry industry^2^. Eggshells can be visually divided into a membrane and a calcified part, and the structure of the latter determines the main properties of avian eggshell. Using a high-resolution electron microscope, Heyn confirmed the structure of eggshell and described this hierarchical structure of eggshell in greater detail than previously. He suggested that the mammillary layer consisted of well-shaped, small calcite crystals in a random orientation and that the palisade layer (termed the crystalline layer) was composed mainly of closely packed calcite crystals, showing crystallographically controlled cleavage patterns in the fracture surface^3^. X-ray diffraction studies on the crystalline structure of eggshell showed that avian eggshell was composed of calcium carbonate with a calcite modification and that the orientation of these calcite crystals in the palisade layer was almost perpendicular to the shell surface, with a slight incline of 16 to 28 degrees^4^.

The calcified shell is formed in uterine fluid over a period of approximately 20 h, and the entire process consists of three stages: an initial stage, a rapid deposition stage, and a final stage^5^. Mammillae are formed during the initial stage and grow at the nucleation sites on the eggshell membrane when the egg migrates into the red isthmus. Then, the egg undergoes the rapid stage of calcium carbonate deposition to form the main structure of the eggshell, ending with cuticle secretion at the final stage^6^.

Much valuable bioresearch has been carried out on three aspects: the structure of the eggshell itself, the regulation of ions in the uterus and the composition of matrix proteins in the eggshell and uterine fluid. Using microarray analysis, Jonchère *et al.*^7^,^8^ and Brionne *et al.*^9^ characterized the chicken uterus transcriptome in detail and identified a large number of genes that encode matrix proteins or uterine ion transporters. Proteomic analyses of uterine fluid and eggshell matrix identified more than 600 proteins^10^,^11^,^12^. Through candidate gene association analysis, Dunn *et al.* found that the variations in ovalbumin, ovocleidin-116 and RARRES1 were associated with eggshell thickness and mammillary layer thickness^13^. Nevertheless, these discoveries were still far from providing an understanding of the genetic bases of eggshell ultrastructure at a genome-wide level. To achieve this goal, a genome-wide association study (GWAS) was proposed as a promising and powerful tool to discover the genetic bases of phenotypes^14^.

A higher-density SNP platform, the 600 K Affymetrix Chicken SNP array, was recently developed^15^ and is more powerful for revealing the causal mutations or genetic mechanisms underlying eggshell ultrastructure than previous SNP platforms. In the present study, we conducted univariate and conditional GWASs on eggshell ultrastructure traits using the 600 K Affymetrix Chicken SNP array in an F_2_ chicken population comprising a total of 927 66-week-old hens. We also explored the architecture of eggshell ultrastructure using the genome partitioning method. The aim of our work was to uncover the genetic architecture of eggshell ultrastructure and to identify candidate mutations, which may be valuable for the genetic improvement of eggshell quality and furthering our understanding of eggshell biomineralization.

## Results

### Phenotypic description and genetic parameters

**Figure 1:**
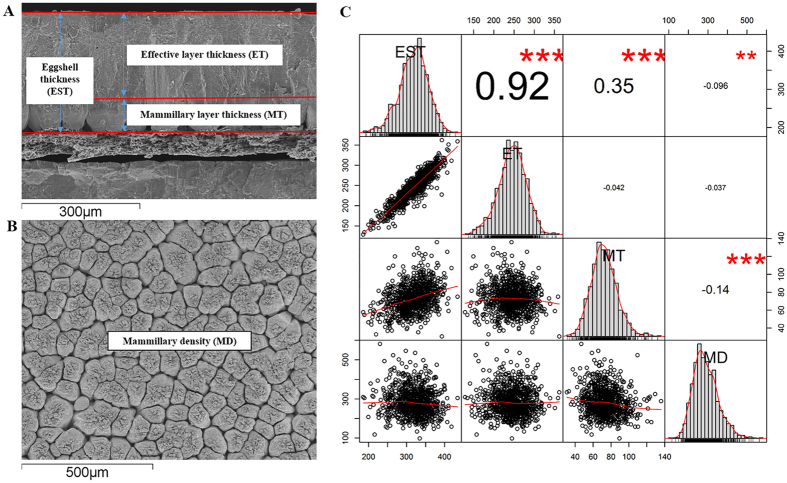
Illustration of eggshell ultrastructure traits (**A,B**) and the correlation matrix plots among them (**C**). EST, ET, MT and MD represent eggshell thickness, effective layer thickness, mammillary layer thickness and mammillary density, respectively. On the diagonal are the univariate distributions, plotted as histograms and kernel density plots (**C**). On the right of the diagonal are the phenotypic pair-wise correlations, with red stars indicating significance levels (***p ≤ 0.001, **0.001 < p ≤ 0.01, *0.01 < p ≤ 0.05). On the left side of the diagonal is the scatter-plot matrix, with LOESS smoothers in red to illustrate the underlying relationship.

Eggshell ultrastructure measurements are illustrated in Fig. 1. The measurements exhibited nearly normal distributions and relatively low phenotypic correlations, except for those between eggshell thickness (EST) and effective layer thickness (ET). Both mammillary layer thickness (MT) and mammillary density (MD) had much higher phenotypic variations (20~24%) than did EST and ET (12~15%) (Table 1). The SNP-based heritabilities (*h*^2^_*snp*_) were moderate for EST and ET (0.36~0.39) and low for MT and MD (0.17~0.19) (Table 2). The absolute values of the genetic correlation were all higher than those of the phenotypic correlation.

### Identification of significant loci by GWAS

**Figure 2:**
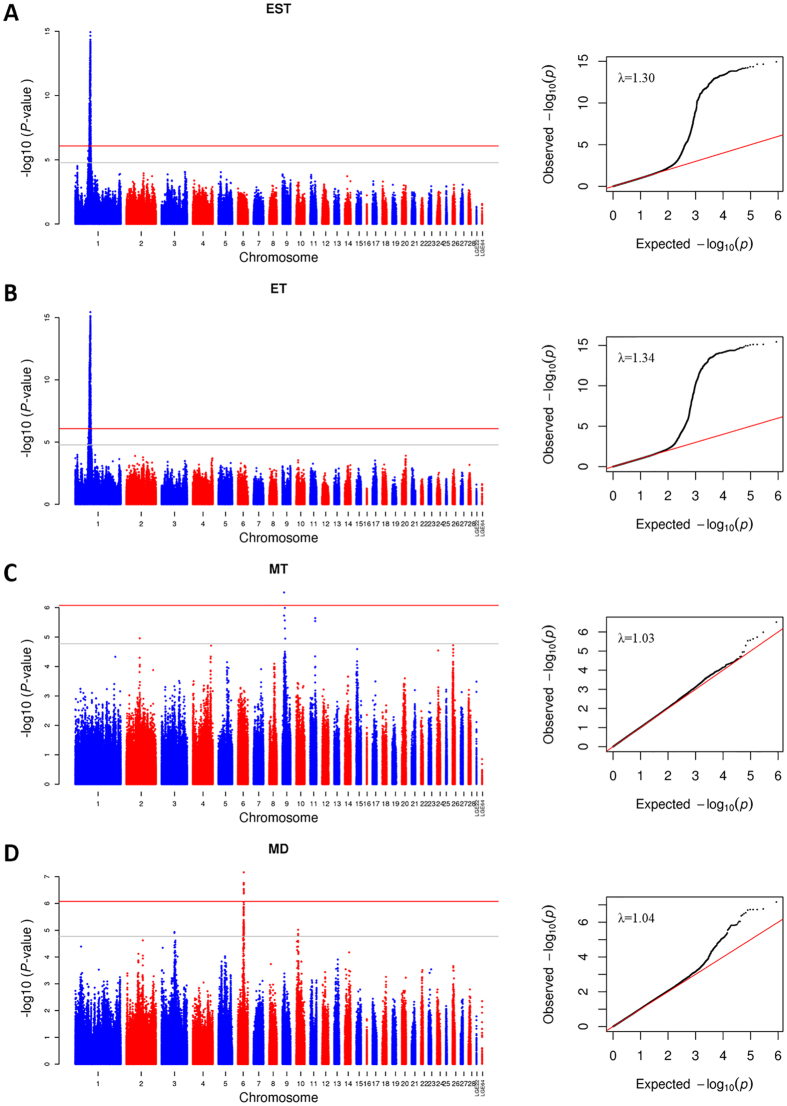
Manhattan plots (left) and quantile–quantile plots (right) of the observed P-values for EST (**A**), ET (**B**), MT (**C**) and MD (**D**). The Manhattan plots indicate -log10 (observed P-values) for genome-wide SNPs (y-axis) plotted against their respective positions on each chromosome (x-axis), and the horizontal gray and red lines depict the genome-wide suggestive (1.71 × 10^−5^) and significant (8.57 × 10^−7^) thresholds, respectively. For quantile-quantile plots, the x-axis shows the expected -log10-transformed P-values, and the y-axis represents the observed -log10-transformed P-values. The genomic inflation factors (λ) are shown on the top left in the QQ plot.

**Figure 3:**
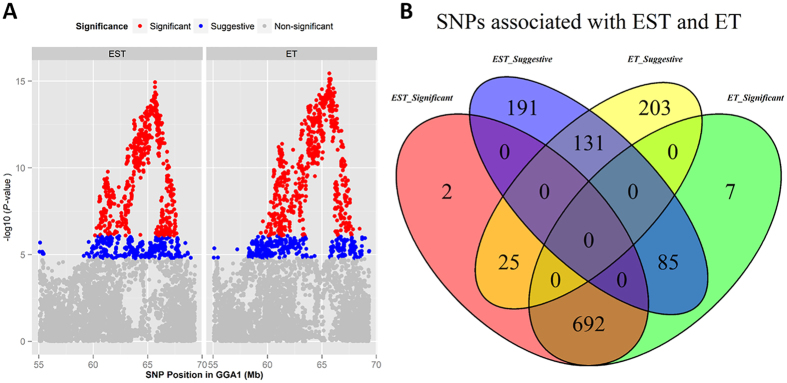
Regional plots (**A**) and Venn diagram (**B**) of significant SNPs associated with EST and ET. The SNPs above the significant (8.57 × 10^−7^) threshold are red, those between the suggestive (1.71 × 10^−5^) and significant threshold are blue, and those below the suggestive threshold are gray.

**Figure 4:**
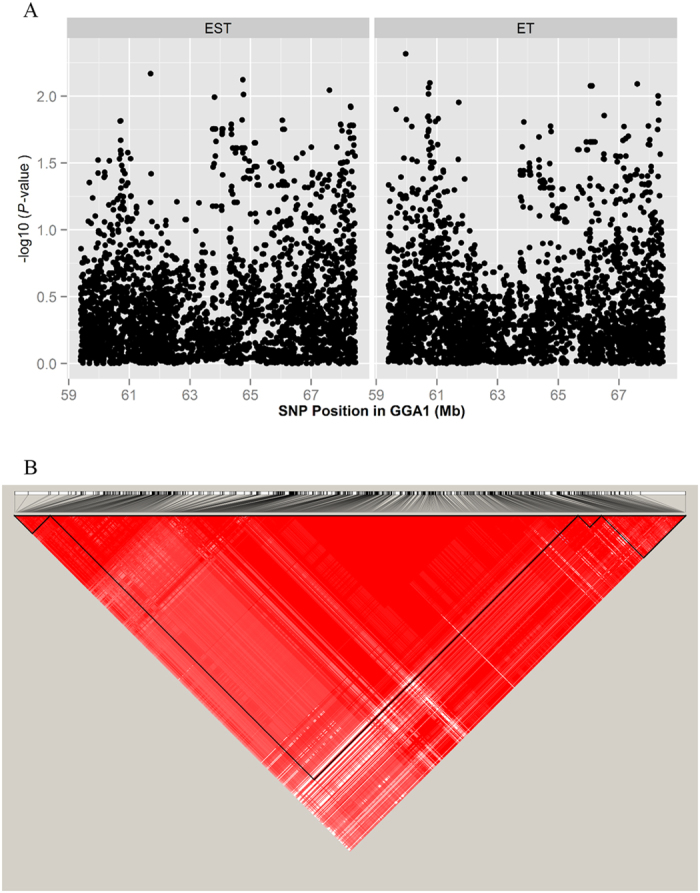
Conditional association analyses of EST and ET (**A**) and linkage disequilibrium (LD) analysis of loci in the significant region associated with EST and ET (**B**). Conditional GWA analyses were performed by fitting the SNPs with minimum P-values as covariates. The strong LD block is defined as D’ ≥0.8.

The univariate analyses revealed a total of 719, 784, 1 and 10 genome-wide significant SNPs associated with EST, ET, MT and MD, respectively (Supplementary Dataset 1). Additionally, 407, 359, 8 and 60 SNPs exhibited associations with those four traits under suggestive level of significance (Supplementary Dataset 2). The Manhattan and quantile-quantile (QQ) plots in Fig. 2 show a global view of the P-values for all SNPs affecting eggshell ultrastructure traits. All significant SNPs associated with EST and ET were located in a 9.1 Mb region spanning from 59.4 Mb to 68.5 Mb on chromosome 1 (GGA1). Owing to the high genetic correlations, EST and ET presented similar P-value distributions of the significant SNPs (Fig. 3A), and as many as 933 of the 1336 SNPs over the suggestive threshold (1.71 × 10^−5^) were overlapping (Fig. 3B). One single SNP that was significantly associated with MT was located at 4.9 Mb on chromosome 9 (GGA9). The 10 SNPs associated with MD were in the region of 20.6~21.4 Mb on chromosome 6 (GGA6). The levels of significance for all loci around the most significant SNP were substantially decreased (Fig. 4A, Supplementary Figure S1) in the stepwise conditional GWA analysis, and no other independent loci were detected. The general and strong linkage disequilibrium (LD) status of loci in this region are visualized in Fig. 4B and Supplementary Figure S2, and the results agree with those of the conditional GWA analysis.

### Annotation of significant SNPs and screening of key genes affecting eggshell ultrastructure

Detailed annotations of the SNPs that affect EST, ET, MT and MD are listed in Supplementary Datasets 3–6, respectively. Four missense SNPs, i.e., rs316793137, rs312347405, rs314218674 and rs312660069, were associated with EST and ET. The SIFT prediction was performed in VEP to evaluate these SNPs impact on protein function. Amino acid substitutions with a score less than 0.05 were predicted to affect protein function^16^. The SIFT scores of those four missense variants ranged from 0.09 to 0.61, which indicated that the amino acid changes were tolerated. The significant SNPs related to EST, ET, MT, and MD were mapped on or near 64, 73, 1 and 2 genes. GO annotations of those candidate genes were obtained from Ensembl BioMart (version 0.7) (Supplementary Datasets 7–10). In terms of eggshell mineral composition, four genes that are involved in ion transport were likely to be the key genes affecting EST and ET: ABCC9 (ATP-Binding Cassette, Sub-Family C (CFTR/MRP), Member 9), ITPR2 (Inositol 1,4,5-Trisphosphate Receptor, Type 2), KCNJ8 (Potassium Channel, Inwardly Rectifying Subfamily J, Member 8) and WNK1 (WNK Lysine Deficient Protein Kinase 1). ITM2C (Integral Membrane Protein 2C) was the only gene that was significantly associated with MT, and KNDC1 (Kinase Non-Catalytic C-Lobe Domain Containing 1) was probably associated with MD.

Because the genes associated with EST and ET were numerous, we specifically explored the interrelationships among these genes. However, using the Functional Annotation and Gene Functional Classification tools in DAVID v6.7 software^17^,^18^, these genes were not significantly enriched in any Gene Ontology terms, pathways or functional groups.

### Allelic contribution to phenotypic variation

**Figure 5:**
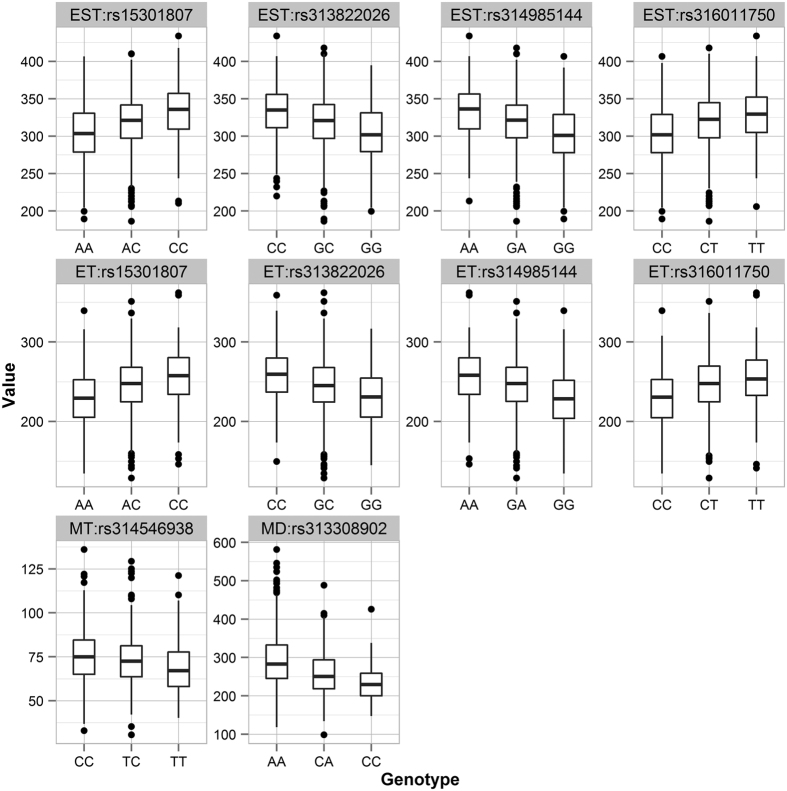
Boxplots of EST, ET, MT and MD versus their corresponding representative SNPs. The x-axis shows genotypes of each SNP. The y-axis represents the phenotypic values of EST, ET, MT and MD. The unit for EST, ET and MD is μm.

The following SNPs in each of the above six genes with a minimum P-value were selected for further analysis: rs15301807, rs313822026, rs314985144, rs316011750, rs314546938 and rs313308902 in KCNJ8, WNK1, ABCC9, ITPR2, ITM2C and KNDC1, respectively. The first four SNPs exerted a 3.2~6.3% contribution to the phenotypic variation of EST and ET. The SNP rs314546938 explained 3.34% of the variation in MT, and rs313308902 contributed 3.91% of the variation in MD (Table 3). Notably, rs314985144 exhibited the largest allelic substitution effect on EST and ET. The substitution of one copy of the effect allele at the rs314985144 site would cause a 0.361 and 0.366 SD/allele (13.98 μm and 13.28 μm) decrease in EST and ET, respectively. In contrast, for rs313308902, such a substitution would result in a 30 mammilla/mm^2^ decrease in MD, which would account for 10% of the average mammilla density of the eggshell. The phenotypic differences among genotypes of those SNPs are shown in boxplots (Fig. 5), revealing that the homozygotes of the effect allele and of the alternative alleles possessed the lowest and highest phenotypic values, respectively, whereas the heterozygotes were intermediate.

### Genome partitioning of genetic variation

**Figure 6:**
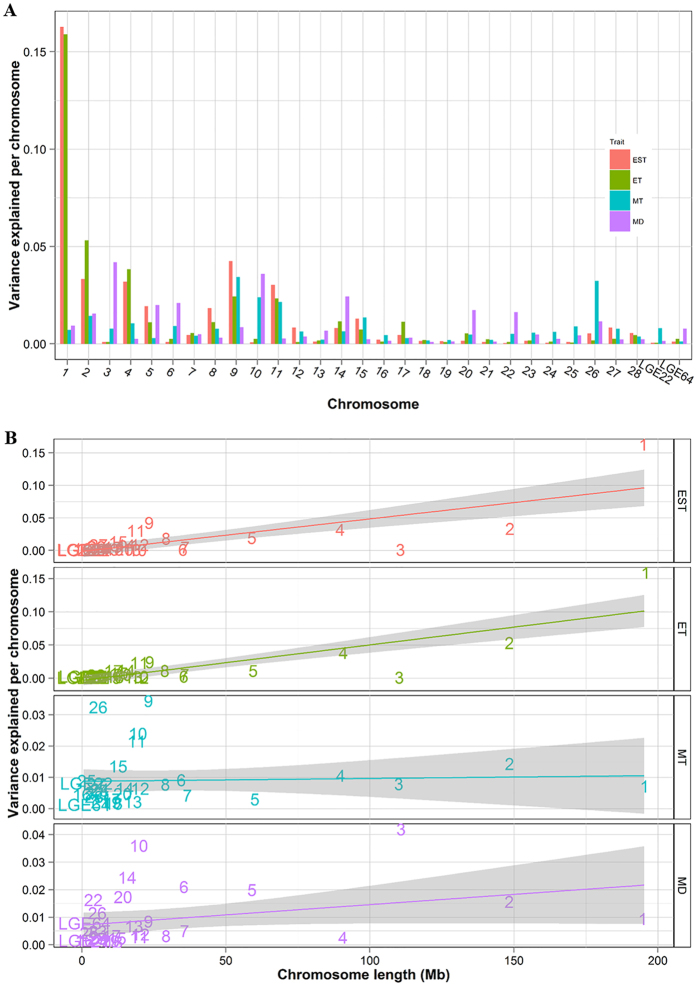
Genome partitioning of variance in EST, ET, MT and MD. **(A)** Contributions of GWAS SNPs partitioned by chromosome. The bars indicate the estimates of variance explained by each chromosome. **(B)** The linear relationships between the variance explained by each chromosome and chromosome length were significant for EST (R^2^ = 0.57, *p* = 9.6e–7) and ET (R^2^ = 0.67, *p* = 1.9e–8), but not for MT and MD. When the GGA1 data were eliminated from the calculations, the linear relationships were weakened but still significant for EST (R^2^ = 0.23, *p* = 5.1e–3) and ET (R^2^ = 0.49, *p* = 1.5e–5).

By partitioning the genetic variances to chromosomes, we dissected the genetic architecture of eggshell ultrastructure. GGA1 was the major effect chromosome for both EST and ET and explained 16.3% and 15.9% of the variance, respectively (Fig. 6A). The chromosomes with maximum effects on MT and MD were GGA9 and GGA3, which explained 3.4% and 4.2% of the variance, respectively. Moreover, we explored the relationship between chromosome length and the corresponding genetic variance explained per chromosome (Fig. 6B). There were clear linear relationships between both EST and ET and chromosome lengths (Adjusted R^2^: 0.5668, p: 9.591e–07; Adjusted R^2^: 0.6709, p: 1.907e–08) but not for MT and MD (Adjusted R^2^: −0.03309, p: 0.7916; Adjusted R^2^: 0.07648, p: 0.07574).

## Discussion

Although the ultrastructure of eggshell was defined several decades ago through the use of advanced techniques such as high-resolution electron microscopes, the genetic mechanism that determines eggshell ultrastructure and the phenotypic and genetic relationships among ultrastructure traits have remained unclear. Thus, we performed GWA analyses in an F_2_-segregated population by utilizing high-density 600 K Affymetrix Chicken SNP arrays. To the best of our knowledge, this is the first GWA analysis of eggshell ultrastructure traits other than EST, and both the large population and the high-density SNP arrays ensured that the results were accurate and reliable. The ET and MT in our study (244.65 μm and 73.5 μm) were slightly thinner than those reported by Dunn *et al.*^13^ (280 μm and 85 μm), but surprisingly, the average ratios of MT to ET in current study and Dunn *et al.*^13^ were both 0.30. The higher heritabilities and lower coefficients of the variation of EST and ET than of MT and MD may be partly due to the vulnerability of mammillae to environmental factors during their short formation time, which is also consistent with their relatively low heritabilities. Interestingly, we found that mammillary layers with denser mammillae tended to be thinner, but there was no obvious effect on EST or ET (Fig. 1C), which was inconsistent with previous studies^19^,^20^. A two-dimensional computer simulation study of layered crystal aggregates suggested that the crystals grew by competing for spaces and that the degree of preferential orientation in the outer part of the crystal aggregate depended mostly on the nucleation density and thickness of the aggregate^19^. Another hypothesis about the interaction between crystal growth and eggshell structure was that mammillary density affects mammillary fusion and, as a consequence, the potential thickness of the mammillary layer^20^. However, eggshell X-ray diffraction studies showed a huge structural difference between the mammillary and effective layers: the crystal orientation of the mammillae was random, as found in powdered eggshell and calcite, but that of the effective layer was oriented to a few crystal planes^4^. Therefore, we hypothesized that differences in crystal structure result in the mammillae having a weak effect on the effective layer. However, the severe defects in the mammillae did have negative effects on eggshell quality^21^,^22^. Apart from these defects, there was no evidence that MD affected ET or EST. We therefore suggested that the mammillae being formed were not just elongated around and upward in a simple way but that as the beginning of the entire eggshell formation process, they needed more complex regulation and the cooperation of various factors.

Association analyses of eggshell ultrastructure traits were conducted separately. The distribution of the resultant P-values on a -log_10_ scale displayed a serious leftward skew for EST and ET and a moderate skew for MT and MD (Fig. 2), which clearly suggested an underlying polygenic architecture^23^. The conditional GWA analysis of these SNPs showed no independent loci (Fig. 4A), and LD analysis revealed that the SNPs in this region were closely linked together (Fig. 4B), which may have been due to insufficient recombination in this region in the F_2_ population. To further reveal the polygenic features of eggshell ultrastructure, we estimated the genetic variance explained by each chromosome (Fig. 6A). Overall, the estimated effects of the chromosomes were consistent with the results of GWA analyses, and the chromosomes that had relatively large effects possessed most of the significant SNPs, which was accurate for EST, ET and MT. These results indicated that our mapped loci or regions played major roles in the formation of different layers of the chicken eggshell. However, the effect of GGA6, which possessed all of the significant SNPs associated with MD, was far lower than those of GGA3, GGA10 and GGA14. This result suggested that the major effect loci were in GGA6 but that numerous minor effect loci were scattered mainly in GGA3, GGA10 and GGA14. Clear linear relationships between the chromosome lengths and the contributions to the genetic variance of EST and ET (Fig. 6B) were consistent with most of the other traits^24^,^25^,^26^. Although these relationships were usually explained by the fact that longer chromosomes are likely to contain more effective markers^25^, this was not always the case. There were no linear relationships between chromosome lengths and the explained variance in MT and MD (Fig. 6B). These two types of cases could be considered two types of polygenic patterns, and further studies are needed to illustrate them in detail.

It is also interesting to note that the significant region (59.4 Mb to 68.5 Mb in GGA1) associated with EST and ET overlapped with the region that Sun *et al.* reported to be associated with eggshell quality^27^. The numbers of SNPs associated with eggshell quality varied with age, but the SNPs identified at 60 to 66 weeks of age accounted for most of the total across all ages^27^. The eggshell ultrastructure traits were measured at 66 weeks of age in the current study. Therefore, we believed that we had uncovered most of the potential SNPs affecting eggshell ultrastructure traits.

In this study, the ABCC9, ITPR2, KCNJ8 and WNK1 genes were associated with EST and ET, and these genes are all involved in ion transport and their expression has been detected in the uterus (Li *et al.* in publication). Previous studies have demonstrated that ion transport plays vital roles in eggshell formation^8^,^28^. We speculated that more than four genes likely affected EST and ET by regulating ion transport and eggshell mineralization. In particular, the combination of ABCC9 and KCNJ8, which encode two different types of ATP-sensitive potassium channel (K_ATP_) subunits, is worthy of greater attention. The K_ATP_ channel is distributed in a variety of tissues^29^. In different tissues, the specific Kir6.x and SURx subunit combinations and their biophysical and pharmacological properties differ markedly^30^. The molecular function of K_ATP_ channels involves the electrical activity of cell membranes in response to cellular metabolism^30^, and channel opening and closing occur in response to intracellular changes in the ADP/ATP ratio^31^. The inhibition of the K_ATP_ channel would lead to an increase in intracellular Ca^2+^ via the Ca^2+^ influx and intracellular Ca^2+^ release that result from membrane depolarization and, thus, the activation of voltage-dependent Ca^2+^ channels^32^. Functional research on the K_ATP_ channels in avian uterine cells is lacking. Considering the significant association between K_ATP_ channels and EST and ET, we hypothesized that K_ATP_ channels exert a role in controlling uterine intracellular and extracellular Ca^2+^ flux and concentration and thus regulate eggshell mineralization.

The ITPR1 gene family has been found to affect eggshell thickness in a previous study by our group^28^. In current study, ITPR2, the type II isoform, was associated with EST and ET. To date, five distinct isoforms of ITPR genes have been identified, and their expression profiles vary among different tissues^33^. ITPR plays a vital role in various cellular and physiological processes, such as cell division, cell proliferation, apoptosis and development^34^. ITPRs are tetrameric intracellular Ca^2+^ channels and are involved in Ca^2+^ release from both secretory vesicles^35^ and the Golgi apparatus^36^. Concurrently, the function of ITPRs is regulated by Ca^2+^ and inositol 1,4,5-trisphosphate (IP3)^37^. The concentration of cytosolic Ca^2+^ could increase rapidly as IP3 binds to ITPR within the membrane of organelles that function as intracellular Ca^2+^ stores. The oscillating mobilization of Ca^2+^ from internal stores is believed to involve the complex interplay between changes in the IP3 concentration and feed-back regulation of ITPR^38^. In many cells, the endoplasmic reticulum (ER) may be the main source of intracellular Ca^2+^ stores^39^, which are released in response to the intracellular signals that are transmitted by extracellular stimuli^37^. ITPR2 was found to be overexpressed in the avian uterus compared to in the magnum^8^ and was associated with eggshell strength (ESS)^27^. We propose that ITPR2 is involved not only in Ca^2+^ signal transduction in uterine cells but also in Ca^2+^ release into the cytosol, most likely from the ER, which provides the necessary Ca^2+^ for eggshell formation. Furthermore, the timing and duration of the ITPR2 channel being open may control the amount of Ca^2+^ deposited into the eggshell and thus regulate eggshell thickness.

WNK1 encodes a member of the WNK subfamily of serine/threonine protein kinases, which are expressed in various tissues, such as the testis, heart, kidney, and skeletal muscle and brain^40^. There is few information concerning the roles of WNK1 in eggshell formation or any other physiological activities of the avian uterus. Here, we found that WNK1 affects EST and ET. Previous studies have shown that epithelial sodium channels are involved in eggshell formation and that their mutations are associated with EST or ESW (eggshell weight)^28^,^41^. Xu *et al.* revealed that WNK1 increases epithelial sodium channel activity by activating PI3 kinase and stimulating the epithelial sodium channel regulator SGK1^42^. Thus, the effect of WNK1 on EST and ET may be exerted by its regulation of epithelial sodium channels. However, this may not be the only pathway because the ion transport systems of eggshell formation involve various ions channels^7^,^8^,^9^, some of which are regulated by WNK1, including the Na^+^-Cl^−^ cotransporter, Na^+^-K^+^-2Cl^−^ cotransporter, K^+^-Cl^−^ cotransporter, K^+^ channel and Na^+^ channel^43^. We suggest that WNK1 has an indirect effect on EST and ET by regulating the activity of epithelial sodium channels and (or) other channels in ion transport systems of eggshell formation.

Mammillae are the narrow round tips of the calcitic cones, which form in the initial stage of eggshell mineralization and are the first layer of the eggshell. According to the hypothesis of competitive growth between adjacent nucleation sites, mammillae grow outwards from the nucleation sites on eggshell membrane and gradually come together and fuse because of limited space^19^. Thus, the mammillary layer thickness (MT) would be determined by the lateral space and the amount of calcium supplied for mammilla growth. The lateral space is restricted by the mammillary density. The results presented in Fig. 1C show that the denser mammillae tended to be thinner. The present study also demonstrated that a significant association existed between ITM2C and MT. The protein-protein interaction data in the STRING database (http://string-db.org) show that ITM2C interacted with RIT2 (Ras-like without CAAX2)^44^, which played a role in regulating the signal transduction cascades by controlling the assembly of protein signaling complexes^45^. RIT2 was found to strongly bind to calmodulin in a calmodulin binding assay^46^. The protein calmodulin regulates a large number of enzymes, ion channels, and other proteins and is involved in various cellular processes^47^. It is worth noting that calmodulin is involved in the inhibition of ITPR by Ca^2+^, the suggested mechanism of which was that “one lobe of calmodulin tethers it to the IP3 receptor, while the other lobe can bind Ca^2+^ and then interact with a second site on the receptor to cause inhibition”^37^. The role of ITPR in Ca^2+^ transport was discussed above. Taken together, these results indicate that MT is affected by mammillary density and might be regulated by ITM2C through the protein interactions of ITM2C-RIT2-Calmodulin-ITPR.

As mentioned previously, mammillae grow from the nucleation sites on the eggshell membrane. This begins in the red isthmus, not the uterus, after the formation of the membrane and the fiber cores, which are the nucleation sites responsible for initiating eggshell mineralization^6^. The density of mammillae (MD) is determined by the nucleation sites, or fiber cores, on the eggshell membrane. However, fiber core studies have been limited. In this study, we found that KNDC1 was significantly associated with MD. The Kinase Non-Catalytic C-Lobe Domain has been shown to be a putative protein-protein interaction module^48^. KNDC1 has been demonstrated to interact with the high-molecular weight microtubule associated protein 2, which leads to the negative regulation of neuronal dendrite growth^49^,^50^. GO annotations showed that KNDC1 was involved in both small GTPase-mediated signal transduction and protein phosphorylation (Supplementary Dataset 10). Based on the above findings, we suggest that KNDC1 may be involved in fiber core protein modification, which determines whether a fiber core will be a nucleation point. The precise functional role of KNDC1 on MD remains to be elucidated. We proposed two ways to illustrate the function of KNDC1: a direct way, in which KNDC1 directly interacts with the fiber core proteins, and an indirect way, in which KNDC1 modifies fiber core proteins through its involvement in signal transduction. In summary, the major effect loci that affect eggshell ultrastructure were located in the region spanning 59.4 Mb to 68.5 Mb in GGA1 for ET and EST, 20.6 Mb to 21.4 Mb in GGA6 for MD and at approximately 4.9 Mb in GGA9 for MT. Four candidate genes, ABCC9, KCNJ8, ITPR2, and WNK1, were considered to be associated with ET and EST on the basis of their regulatory roles in the uterus ion transport system. MT was determined by both the physical space around mammillae, which is affected by mammillary density, and the physiological process of calcium carbonate deposition, which is regulated by the protein interactions of ITM2C-RIT2-Calmodulin-ITPR. MD may be regulated by the KNDC1-mediated modification of fiber core proteins at nucleation sites either directly or indirectly. The present study elucidates the genetic basis of eggshell ultrastructure and contributes to both improving eggshell quality and our understanding of the ultrastructure and biomineralization of chicken eggshell, as well as other biomineralized materials.

## Materials and Methods

### Ethics statements

All blood samples were collected from brachial veins of chickens by standard venipuncture. The whole procedure was performed according to regulations and guidelines established by the Animal Care and Use Committee of China Agricultural University. The entire study was approved by Animal Care and Use Committee of China Agricultural University (permit number: SYXK 2007–0023).

### Experimental population

White Leghorn (WL) and Dongxiang chickens (DX), representing a standard breed and a Chinese indigenous strain, respectively, were used to construct an F_2_ resource population. First, the mating of six WL males with 133 DX females and six DX males with 80 WL females produced an F_1_ generation of 1,029 and 552 birds, respectively. Then, 25 cocks and 407 hens from the WL/DX cross and 24 cocks and 235 hens from DX/WL cross in the F_1_ generation were selected to produce the F_2_ generation. The F_2_ population consisted of 1,893 hens and 1,856 cocks from 49 half-sib and 590 full-sib families in the same hatch. To ensure sufficient phenotypic information and accurate pedigree, 927 hens from 49 half-sib families and 365 full-sib families were selected for SNP genotyping.

### Phenotypic measurements

The ultrastructure of eggshell in the current study refers to eggshell thickness (EST), effective layer thickness (ET), mammillary layer thickness (MT), and mammillary density (MD) (Fig. 1). EST is the thickness of the total calcified shell. ET is the combined thickness of the palisade, vertical crystal and cuticle layer, as defined by Dunn *et al.*^13^. MT is the thickness of the mammillary layer. MD is the number of mammillae per square millimeter. Eggs, one per hen, were collected at 66 weeks of age. The egg contents were removed, and the eggshells were cleaned with tap water and dried naturally at room temperature. Two shell slices with a size of 3 mm × 5 mm were taken at the equator of each eggshell. One slice was used for eggshell cross section observations, and the other was boiled in 3% sodium hydroxide solution for 10 minutes to dissolve the membrane covering the mammillae and was then dried at room temperature for subsequent observation of the eggshell mammilla numbers. The prepared samples were measured using a scanning electron microscope (JEOL JSM-6301F, Japan). The thickness of each layer and the number of eggshell mammillae were measured using the ImageJ software (http://rsb.info.nih.gov/ij/). Fewer than half of the mammillae, as judged by the naked eye, were omitted. The area of mammillary images was calculated as 1.02 mm^2^, according to the scale. Descriptive statistical analyses were conducted in the R environment (R version 3.0.2) using all available records. The traits, deviating from normality, were assessed by rank-based inverse normal transformations using the ‘rntransform’ function in the GenABEL package of R^51^.

### Genotyping, quality control (QC) and imputation

Genomic DNA was extracted from blood samples using standard phenol-chloroform method and quantified using a NanoDrop spectrophotometer (GE Healthcare Life Sciences, Uppsala, Sweden); the final concentrations were 30~50 ng/μl. Genotyping was conducted on a 600 K Affymetrix Axiom Chicken Genotyping Array (Affymetrix, Inc. Santa Clara, CA, USA). The quality control and genotype calling were implemented using the Affymetrix Power Tools v1.16.0 (APT) software with the Axiom GT1 algorithm. Only samples with a dish quality control (DQC) >0.82 and a call rate >97% were included in the subsequent analyses. The SNP QC metrics were calculated using an R script supplied by Affymetrix with the default parameters; individual SNPs that fell below the given thresholds were filtered out. Finally, 927 samples and 532,299 SNPs remained valid after the above QC steps. Considering the relatively low power of detection of the associations between phenotypes and sex chromosome genotypes for the current statistical methods, the SNPs on sex chromosomes were excluded. Moreover, the SNPs with a minor allele frequency (MAF) <5% and a Hardy-Weinberg equilibrium (HWE) test P < 1 × 10^−6^ were removed using the PLINK v1.90 package^52^. The BEAGLE v4.0 procedure was used to impute sporadic missing genotypes^53^, and the SNPs with an imputation quality score R^2^ > 0.5 were retained for the next analysis step. Finally, a total of 927 samples and 434,442 SNPs remained for the subsequent GWASs.

### Association analysis

The statistical model used for GWA analysis was a linear mixed model. The first five principal components (PCs) calculated using the independent SNPs were considered covariates in the mixed model to eliminate spurious associations due to the presence of potential cryptic relatedness or hidden population stratification. Considering the over-conservation of Bonferroni correction, we adjusted the genome-wide significant threshold using the simpleM method^54^. The genome-wide suggestive and significant P-values were 1.71 × 10^−5^ (1.00/58,358) and 8.57 × 10^−7^ (0.05/58,358), according to the effective number (58,358) of independent tests, as calculated using the simpleM method. The GEMMA v0.94 software was used to test the associations of all valid SNPs and each trait with the mixed model^55^. The Wald test P-value was selected to measure the significance between SNPs and traits. The Manhattan plots and quantile-quantile (QQ) plots were generated using the “gap” (Version 1.1–16) and “qqman” (Version 0.1.2) packages in R. To judge the extent of false positive signals, the genomic inflation factor λ was calculated using the GenABEL package^51^ in R. Stepwise conditional analyses were conducted using the same mixed model with the addition of the strongest SNP identified in the above association analysis as a covariate^56^.

### Estimation of variance explained

We estimated the pair-wise genetic and phenotypic correlations among eggshell ultrastructure traits using the bivariate mixed model and *h*^2^_*snp*_ using the restricted maximum likelihood (REML) method in the GCTA v1.24 program^57^. For the genome-wide significant SNPs or regions, we calculated their contributions to the phenotypic variances (CPVs). Furthermore, we estimated the CPVs of each chromosome and linkage group for the traits of interest^25^,^56^.

### Linkage disequilibrium (LD) analysis and gene identification

We conducted LD analyses and inferred the haplotype blocks containing peak SNPs by the solid spine algorithm in Haploview v4.2 software^58^. The strong LD block was defined as the SNPs at two ends in a region being in strong LD (D’ ≥0.8) with all intermediate SNPs. The significant SNPs were functionally annotated, and the candidate genes related to significant SNPs or genomic regions were identified using Variant Effect Predictor (VEP)^59^ and Biomart tools^60^ supported by Ensembl based on the Galgal4 assembly.

## Additional Information

**How to cite this article**: Duan, Z. *et al.* Genetic architecture dissection by genome-wide association analysis reveals avian eggshell ultrastructure traits. *Sci. Rep.* **6**, 28836; doi: 10.1038/srep28836 (2016).

## Supplementary Material

Supplementary Information

Supplementary Dataset 1

Supplementary Dataset 2

Supplementary Dataset 3

Supplementary Dataset 4

Supplementary Dataset 5

Supplementary Dataset 6

Supplementary Dataset 7

Supplementary Dataset 8

Supplementary Dataset 9

Supplementary Dataset 10

## Figures and Tables

**Table 1:** Descriptive statistics for eggshell ultrastructure traits.

Trait^a^	N	Mean	SD	CV (%)	Min	Max	Ratio of each layer to EST (%)
EST (μm)	927	318.33	38.73	12.17	186.32	433.81	100.0
ET (μm)	927	244.65	36.28	14.83	128.69	361.98	76.9
MT (μm)	925	73.50	15.02	20.44	30.64	135.98	23.1
MD	913	284.76	67.87	23.83	98.63	581.05	–

Abbreviations: N = number of samples; Mean = arithmetic mean; SD = standard deviation; CV = coefficient of variance; Min = minimum; Max = maximum; Percentage = the average percentage of each layer thickness.

^a^EST = eggshell thickness; ET = effective layer thickness; MT = mammillary layer thickness; MD = mammillary density.

**Table 2:** Genetic parameters for eggshell ultrastructure traits.

Trait^a^	EST	ET	MT	MD
EST	0.39 (0.06)	0.96 (0.02)	0.60 (0.15)	−0.29 (0.16)
ET		0.36 (0.06)	0.35 (0.20)	−0.26 (0.18)
MT			0.17 (0.05)	−0.26 (0.21)
MD				0.19 (0.05)

Diagonal: heritability estimates. Upper triangle: genetic correlations. Standard errors of the estimates are in parentheses.

^a^EST = eggshell thickness; ET = effective layer thickness; MT = mammillary layer thickness; MD = mammillary density.

**Table 3:** Contributions of seven SNPs in genes potentially related to eggshell ultrastructure.

SNP		rs15301807	rs313822026	rs314985144	rs316011750	rs314546938	rs313308902
Gene		KCNJ8	WNK1	ABCC9	ITPR2	ITM2C	KNDC1
Consequence		Intron variant	Intron variant	Intron variant	Intron variant	Downstream gene variant	Downstream gene variant
Chromosome		1	1	1	1	9	6
Position (bp)		67106463	60604387	67055169	67859289	4863515	21070589
EA/AA		A/C	G/C	G/A	C/T	T/C	C/A
MAF		0.472	0.478	0.473	0.453	0.344	0.116
EST	beta (SE)^a^	−0.347 (0.058)	−0.348 (0.059)	−0.361 (0.058)	−0.277 (0.059)	–	–
	CPV (%)	5.52	5.50	6.09	3.23	–	–
ET	beta (SE)	−0.329 (0.056)	−0.365 (0.056)	−0.366 (0.055)	−0.290 (0.057)	–	–
	CPV (%)	4.88	6.16	6.28	3.63	–	–
MT	beta (SE)	–	–	–	–	−0.276 (0.053)	–
	CPV (%)	–	–	–	–	3.34	–
MD	beta (SE)	–	–	–	–	–	−0.440 (0.081)
	CPV (%)	–	–	–	–	–	3.91

Abbreviations: EA = effect allele (minor allele); AA = alternative allele (major allele); MAF = minor allele frequency; CPV = contribution to phenotypic variance (%); EST = eggshell thickness; ET = effective layer thickness; MT = mammillary layer thickness; MD = mammillary density.

^a^beta is the estimated allelic substitution effect per copy of the effect allele (EA) based on an inverse-normal transformed scale under an additive model, expressed in SD unit/allele; SE = standard error of the beta.
