# Supplementary material for: Genetic architecture dissection by genome-wide association analysis reveals avian eggshell ultrastructure traits
Source: Sci Rep. 2016 Jul 26;6:28836. doi: 10.1038/srep28836 (PMC4960555; doi:10.1038/srep28836)

**Supplementary information**

**Genetic architecture dissection by genome-wide association analysis reveals avian eggshell ultrastructure traits**

Zhongyi Duan^1,+^, Congjiao Sun^1,+^, ManMan Shen^2^, Kehua Wang^2^, Ning Yang^1^, Jiangxia Zheng^1*^, Guiyun Xu^1*^

**Figure S1. Conditional association analyses of MD.**

**
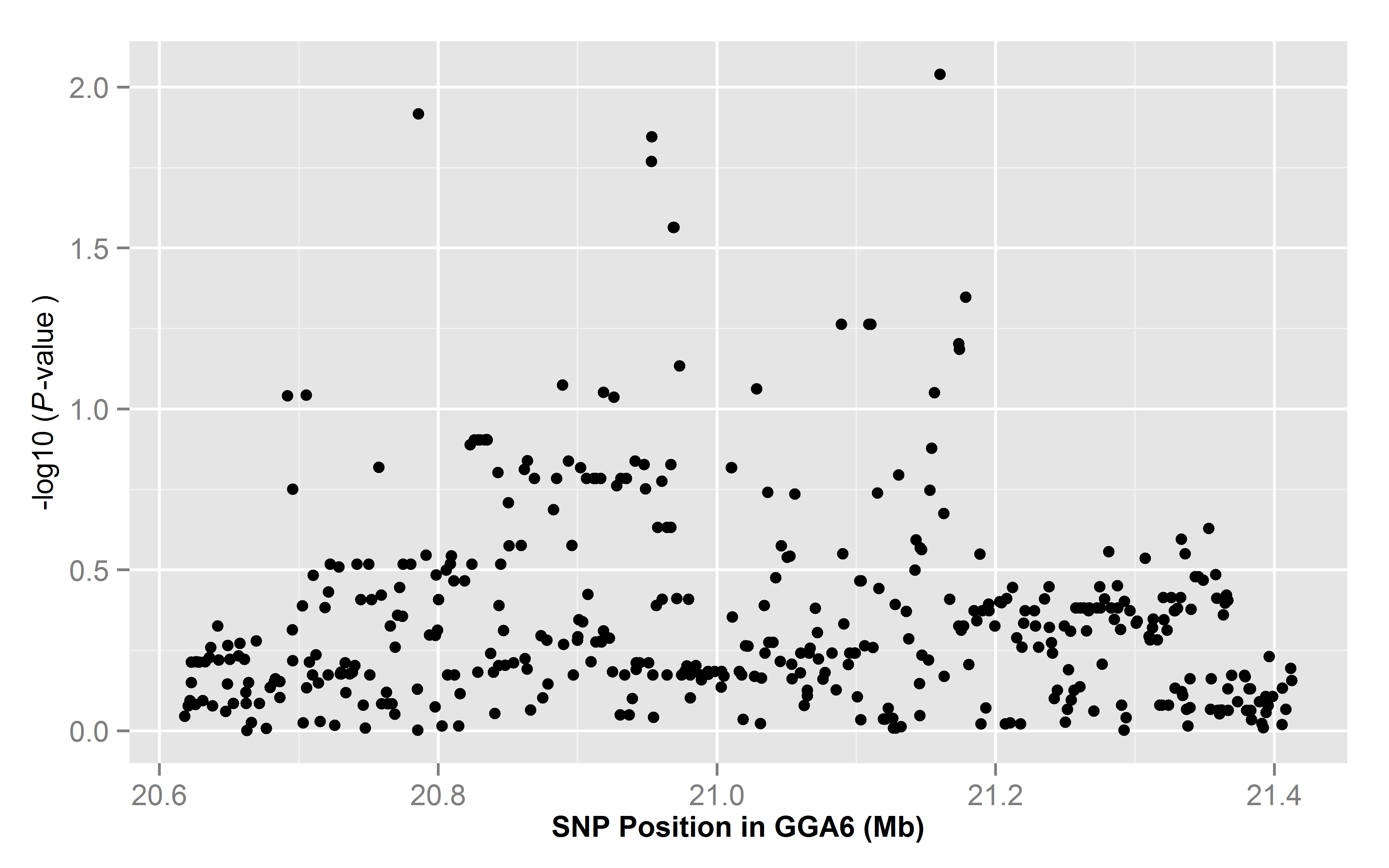
**

**Figure S2. Linkage disequilibrium (LD) analysis of loci associated with MD.** The strong LD block is defined as D’ >= 0.8.


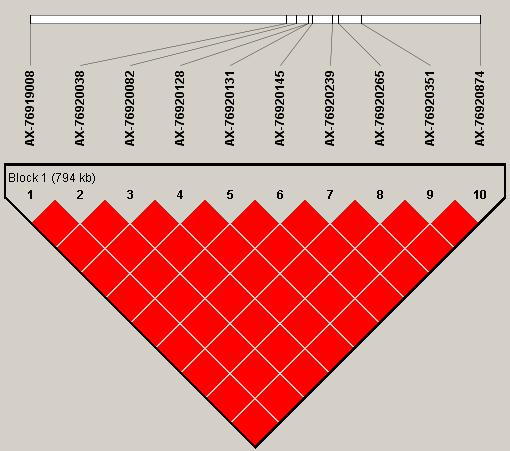

Supplement: Supplementary Information [file srep28836-s1.doc]
